# Black Soldier Fly: A Keystone Species for the Future of Sustainable Waste Management and Nutritional Resource Development: A Review

**DOI:** 10.3390/insects16080750

**Published:** 2025-07-22

**Authors:** Muhammad Raheel Tariq, Shaojuan Liu, Fei Wang, Hui Wang, Qianyuan Mo, Zhikai Zhuang, Chaozhong Zheng, Yanwen Liang, Youming Liu, Kashif ur Rehman, Murat Helvaci, Jianguang Qin, Chengpeng Li

**Affiliations:** 1 College of Food Science and Technology, Huazhong Agricultural University, Wuhan 524088, China; raheeltariq916@gmail.com (M.R.T.);; 2 Agricultural Products Processing Research Institute, Chinese Academy of Tropical Agricultural Sciences, Zhanjiang 524001, China; liushaojuan2023@163.com (S.L.);; 3 German Institute of Food Technologies (DIL e.V.), Prof.-v.-Klitzing-Str. 7, 49610 Quakenbrück, Germany; 4 Department of Microbiology, Faculty of Veterinary and Animal Sciences, The Islamia University of Bahawalpur, Bahawalpur 63100, Pakistan; 5 Department of Horticulture, Faculty of Agricultural Sciences and Technologies, European University of Lefke, Gemikonagi, Northern Cyprus, Mersin 99780, Türkiye; 6 College of Science & Engineering, Flinders University, Adelaide 5001, Australia; 7 School of Chemistry and Environment Science, Guangdong Ocean University, Zhanjiang 524088, China

**Keywords:** *Hermetia illucens*, organic waste valorization, insect bioconversion, alternative protein production, insect genomics

## Abstract

The global escalation of organic waste generation, coupled with rising protein demand and environmental pressure, necessitates innovative, circular approaches to resource management. *Hermetia illucens* (Black Soldier Fly, BSF) has emerged as a leading candidate for integrated waste-to-resource systems. This review examines BSF biological and genomic adaptations underpinning waste conversion efficiency, comparative performance of BSF bioconversion versus traditional treatments, nutritional and functional attributes, techno-economic, regulatory, and safety barriers to industrial scale-up. Peer-reviewed studies were screened for methodological rigor, and data on life cycle traits, conversion metrics, and product compositions were synthesized. BSF larvae achieve high waste reductions, feed-conversion efficiencies and redirect substrate carbon into biomass, yielding net CO_2_ emissions as low as 12–17 kg CO_2_ eq ton^−1^, an order of magnitude below composting or vermicomposting. Larval biomass offers protein, lipids (notably lauric acid), micronutrients, chitin, and antimicrobial peptides, with frass serving as a nutrient-rich fertilizer. Pathogen and antibiotic resistance gene loads decrease during bioconversion. Key constraints include substrate heterogeneity, heavy metal accumulation, fragmented regulatory landscapes, and high energy and capital demands. BSF systems demonstrate superior environmental and nutritional performance compared to conventional waste treatments. Harmonized safety standards, feedstock pretreatment, automation, and green extraction methods are critical to overcoming scale-up barriers. Interdisciplinary innovation and policy alignment will enable BSF platforms to realize their full potential within circular bio-economies.

## Simple Summary

The Black Soldier Fly (BSF) presents a sustainable solution to global challenges like food demand, waste management, and environmental degradation. With a short life cycle and robust genetictraits, BSF larvae efficiently convert organic waste into high-protein biomass and nutrient-rich frass for fertilizer. Compared to traditional methods like composting, BSF systems lower greenhouse gas emissions, reduce pathogens, and minimize the presence of antibiotic resistance genes in waste. Beyond waste management, BSF-derived products have diverse applications, including animal feed (replacing fishmeal and soybean meal), bioplastics (produced from chitin), bioremediation (for detoxifying polluted waste), and antimicrobial peptides for medical applications. However, challenges such as regulatory barriers, risks of heavy metal contamination, and scaling costs must be addressed. With advancements in automation, waste pretreatment, and supportive policies, the BSF can play a key role in a circular economy, transforming waste into valuable resources while minimizing environmental impact.

## 1. Introduction

The convergence of pressing global challenges, including the ever-increasing food demand, escalating environmental degradation, and the generation of excessive organic waste, underscores the critical and immediate need for integrated, sustainable solutions for resource management [1,2,3]. Recent studies have shown that traditional waste treatment methods, such as landfilling and incineration, are not only environmentally detrimental but also economically unsustainable [4,5,6,7]. Conventional protein sources like soybean meal and fishmeal, which are directly linked to deforestation and overfishing, raise significant ecological concerns. This underscores the urgent need for alternative solutions [8,9]. This review addresses the following research questions. How can the Black Soldier Fly (BSF) be effectively integrated into sustainable waste management and alternative protein production systems, and what biological, technological, and regulatory challenges must be overcome? The specific objectives are to (a) evaluate the biological and genomic foundations of the BSF that support its efficiency in waste conversion, (b) compare BSF bioconversion, i.e., the process by which BSF larvae convert organic waste into biomass and valuable byproducts with conventional waste treatment methods in terms of environmental impact and byproduct quality, (c) assess the nutritional composition and functional potential of the BSF for use in animal feed and other applications, and (d) identify current scalability, regulatory, and safety challenges. These pressures highlight the need for novel bio-based systems capable of closing nutrient loops, reducing CO_2_ emissions, and enabling the conversion of low-grade waste streams [10,11].

In this context, insects have emerged as a viable biological solution, with the BSF gaining considerable traction as a bioconversion agent and alternative protein source [12,13,14,15,16]. BSF larvae exhibit high feed conversion efficiency and can process a broad spectrum of organic materials, including food waste, agricultural byproducts, and livestock manure [17,18,19]. Through this bioconversion process, the larvae generate valuable biomass rich in proteins, lipids, and micronutrients, while simultaneously reducing the volume and pathogenicity of organic waste [12,18,20,21,22]. Furthermore, the resultant frass has demonstrated efficacy as an organic fertilizer, supporting nutrient recycling and soil health improvement [23,24,25,26,27].

The BSF exhibits a short, well-defined life cycle and broad environmental tolerance. Their rapid adaptability to diverse substrates underpins their significant technological potential in waste management and protein production [28,29,30,31]. Genomic studies reveal an expanded repertoire of detoxification enzymes, immune-related genes, and metabolic pathways associated with amino acid and lipid biosynthesis, all of which facilitate survival and efficiency in challenging environments [32,33,34,35]. Additionally, the larval gut microbiome is dominated by bacteria, which play an important role in waste degradation, nutrient assimilation, and antimicrobial activity, enhancing the system’s biotechnological resilience [36,37].

Beyond nutrient recovery, BSF-derived compounds, such as antimicrobial peptides (AMPs), chitin, and lauric acid, hold promise for applications in agriculture, bioplastics, pharmaceuticals, and functional foods [38,39,40,41,42,43,44]. Recent findings also highlight BSF’s capacity to reduce antibiotic resistance genes or ARGs (ARGs are genetic markers that indicate the presence of antibiotic-resistant bacteria) and pathogens in biosolids, underscoring its value in environmentally safe waste treatment [45,46,47,48].

Despite the above-mentioned features, the industrial scalability of BSF systems is constrained by several factors, including the bioaccumulation of heavy metals, variation in nutritional output based on substrate composition, and insufficient regulatory clarity across regions [22,49,50]. While the BSF offers unique advantages over conventional methods like composting and anaerobic digestion, challenges such as heavy metal bioaccumulation remain unresolved issues, which require emerging microbial treatment technology to address effectively. Moreover, socio-technical challenges such as consumer acceptance, standardization of rearing protocols, and economic viability in resource-limited settings remain unresolved [51,52,53,54,55]. Advancements in automation, genomics, microbial engineering, and waste preprocessing could mitigate these issues, but require coordinated research and policy support [33,56,57,58,59].

This review evaluates the biological capabilities, nutritional value, and diverse industrial applications of *Hermetia illucens*, with particular emphasis on its role in sustainable organic waste conversion and alternative protein production. It further identifies challenges and safety concerns, explores current technological limitations, for research, regulation, and commercialization.

### Methodology

A comprehensive literature search was conducted on the Web of Science, Scopus, and PubMed for articles published between 2001 and 2025. Keywords such as ‘Black Soldier Fly’, ‘*Hermetia illucens*’, ‘waste management’, ‘alternative protein’, and ‘bioconversion’ were used. Only peer-reviewed articles, reviews, and significant case studies written in English were included. The selected studies were evaluated for methodological rigor and relevance to the key themes of this review.

## 2. Biological and Genomic Foundations

### 2.1. BSF Life Cycle

**Figure 1:**
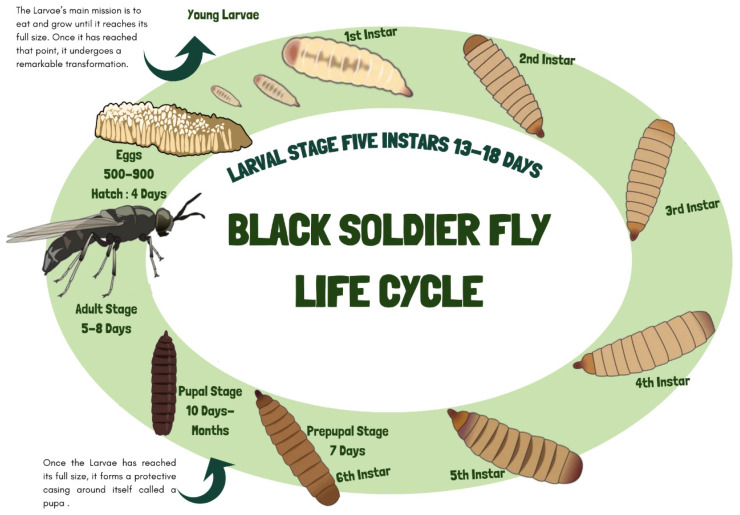
BSF life cycle with each developmental stage.

In the BSF life cycle, the insect progresses from the larval to the prepupal stage and reaches the pupal stage before adulthood. Each stage exhibits distinct morphological and behavioral traits. This insect species has a short development life cycle lasting 45 days with four stages: egg (4 days), larva (13–18 days), prepupa (7–14 days), and adult (5–9 days) [60,61,62]. Figure 1 provides a detailed schematic of the BSF life cycle, indicating the duration of each stage (egg, larva, prepupa, pupa, and adult), along with key morphological changes. The eggs are small (1 mm) and present a color range from white to cream. During the larval stage, which comprises five instars (developmental phases), the BSF undergoes significant morphological changes, such as a color transition to dark brown, before emerging from the substrate as a prepupa [61]. The larvae can thrive in various decaying organic matter due to their adaptive oral structure, rich intestinal microbiota, and high enzymatic activity, which allows them to metabolize molecules such as starch, protein, and lipids [63]. The duration of the larval stage depends on physical conditions and food availability [61]. Their life cycle and nutritional composition can be influenced by the quality and quantity of the rearing substrate [64,65,66]. Environmental factors like temperature and humidity critically influence BSF growth and reproduction, with optimal conditions (27 ± 2 °C) enhancing life cycle efficiency, adult longevity, and fecundity, while substrate moisture of 45–75% is necessary for the larvae to develop into prepupae [40,60,67,68]. After the larval stage, the insect enters the prepupal stage, where it lies motionless while its cuticle is rigidified and becomes rich in calcium salts, forming a dark envelope. In this stage, the larva empties its digestive tract and no longer needs to feed, relying on the nutrients stored during the larval stage [69]. The larvae show varied growth rates depending on diet [70,71,72]. Using the insect in the prepupal stage might have two advantages. The empty digestive tract can reduce the risk of carrying pathogenic microorganisms, and the prepupal migrating behavior eases harvesting in a large-scale rearing system [73]. Waste management insects and their biomass can provide value-added compounds [65,74].

### 2.2. Genomic Insights

The BSF has a genome of 1.01–1.68 Gb, predominantly repetitive sequences (~67%) and gene families essential for septic adaptation, such as immune system factors, cytochrome P450 detoxification enzymes, and olfactory receptors [33,75,76]. It decomposes a variety of organic substrates efficiently due to its 14,000–17,000 protein-coding genes’ metabolic pathways for amino acid biosynthesis, fatty acid metabolism, and glycerol–lipid processing [33,76]. As summarized in Table 1, the BSF exhibits several genetic adaptations, including an expanded repertoire of detoxification enzymes and immune factors critical to survival in a challenging environment. The gut microbiome, dominated by Firmicutes and Proteobacteria, degrades lignocellulose and proteins with host-derived enzymes (e.g., cellulases, proteases) and supports environmental adaptability. Selective sweeps in metabolism, immunity, and development genes show genomic flexibility in rapid evolutionary responses to low-quality diets and domestication pressures [77,78]. This genetic and environmental adaptability makes it valuable in waste conversion, sustainable protein production, and bioremediation [33,44]. Having established robust genetic and physiological mechanisms that enable the BSF to thrive in diverse conditions, the following section explores how these biological traits translate into effective organic waste management and environmental protection.

**Table 1:** Genetic traits of the BSF and their impact on waste conversion and nutritional production.

Factor	Traits	Organism	Description	References
Genetic	Genomic expansion (immune genes, CYP450s, olfactory receptors)	BSF	Enhanced detoxification, pathogen resistance, and detection of decaying matter via expanded gene families.	[33,75]
	Rapid evolutionary adaptation to diet	BSF	Stronger adaptive responses to low-quality diets (e.g., wheat bran) due to selection pressure.	[77]
	Genotype-by-diet interactions	BSF	Genetic strains show varied growth and nutrient composition depending on substrate.	[79]
	Metabolic gene enrichment (amino acid/fatty acid metabolism)	BSF	Efficient conversion of decaying matter into biomass via enriched metabolic pathways.	[76]
Environmental	Diet-dependent midgut adaptations	BSF	Enzyme activity, cell morphology, and nutrient storage adjust to low-quality diets (e.g., fruit/vegetable waste).	[80]
	Microbiome shifts (Firmicutes, Proteobacteria, Actinobacteria)	BSF	Gut microbiota degrades complex organics; composition changes with substrate type.	[81]
	Behavioral response to VOCs	BSF	Odorant-binding proteins detect volatile organic compounds to locate decay.	[82]
	Waste storage conditions	BSF	Refrigeration promotes beneficial yeasts (e.g., *Pichia*), while open storage increases spoilage fungi.	[83]
Morphology/Physiology	Midgut enzyme plasticity	BSF	Transcriptome shifts in digestion/absorption genes under different diets.	[80]
	Fat body metabolism	BSF	Alters lipid/protein storage in response to nutrient availability (e.g., protein-poor diets).	[84]
	Heavy metal tolerance	BSF	Accumulates Cd/Pb but thrives on non-hazardous waste; limited tolerance to extreme pollution.	[85]
Microbiome Interactions	Lignocellulose degradation	BSF	*Corynebacterium* and *Brevibacterium* in residues break down lignin; gut bacteria synergize with host enzymes.	[86,87,88]
	Protein/lipid digestion	BSF	*Pseudomonas* and *Campylobacter* produce proteases/lipases; microbiota–host synergy enhances nutrient extraction.	[89,90]
Applications	Waste conversion strategies	BSF	Pretreatment (e.g., hydrothermal) and microbiota engineering improve bioconversion efficiency.	[44]
	Genetic breeding/CRISPR	BSF	Enhanced traits (e.g., flightlessness, detoxification) via selective breeding or gene editing.	[33,91]
Genome Insights	Genome size (~1.01–1.68 Gb) and complexity	BSF	Large genome with 14,000–17,000 protein-coding genes, repetitive elements (67%), and expanded immune/metabolic gene families.	[76,92]
	Antimicrobial peptides (defensins, cecropins)	BSF and Other Insects	Protect against pathogens in decay-rich environments via membrane disruption; developmentally regulated expression.	[93]

These genomic adaptations underscore BSF’s capacity to efficiently convert diverse organic substrates, supporting its use in sustainable waste management and protein production.

## 3. Environmental Protection and Waste Management

The BSF has a very promising role in sustainable waste management. It leverages a unique digestive system and symbiotic relationship with gut microbiota to convert a variety of organic waste into valuable byproducts. Below, we outline the key processes and advantages of BSF bioconversion in waste treatment, its byproducts, and associated environmental impacts.

### 3.1. BSF Digestive System and Bioconversion Efficiency

**Figure 2:**
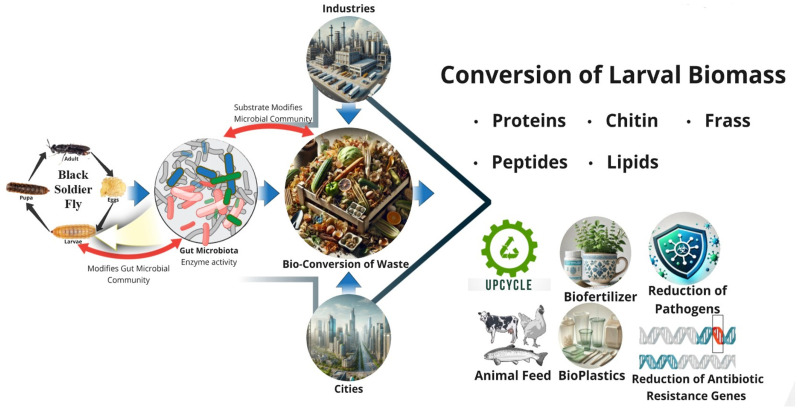
BSF conversion of waste from industries and cities into valuable products, while also exhibiting the influence of substrate on the gut microbiota of the BSF.

BSF larvae exhibit a highly specialized digestive system capable of processing various organic materials. The foregut, midgut, and hindgut work in unison, with the midgut being the primary site for enzymatic digestion, assisted by enzymes such as proteases, lipases, and amylases, along with microvilli that enhance nutrient absorption [94,95,96,97,98]. The BSF also possesses a robust mandibular–maxillary complex that aids in consuming semi-liquid food [99,100,101]. The larvae’s gut microbiota, dominated by bacteria like *Enterococcus*, *Klebsiella*, and *Bacillus*, further contributes to the breakdown of complex organic materials, ensuring efficient nutrient extraction [48,102,103,104,105,106]. The integration of BSF bioconversion processes in urban and industrial waste treatment is depicted in Figure 2, showing how substrate variations influence the gut microbiota of the BSF, thereby affecting conversion efficiency. The bioconversion process allows the BSF to reduce organic waste by up to 84.5%, with feed conversion efficiency reaching 16.15% [107,108,109,110]. While BSF larvae have been reported to reduce organic waste by up to 84.5% and achieve feed conversion efficiencies of approximately 16.15%, these figures are highly substrate- and environment-dependent and should not be treated as universally applicable benchmarks [29,111,112]. Substrate nutritional composition, including crude protein (CP), ether extract (EE), fiber fractions (ADF, NDF), and gross energy (GE), strongly influences larval performance and conversion metrics [103,113,114,115]. Protein-rich substrates such as animal byproducts, fermentation residues, and food waste promote higher biomass yields, bioconversion ratios (BRs), and substrate reduction rates [16,116,117,118]. Whereas, high fiber content or imbalanced nutrient profiles often depress growth and feed conversion [119,120].

Environmental variables also play a pivotal role; elevated rearing temperatures and humidity levels have been shown to enhance fresh larval weight, dry matter conversion, and metabolic efficiency [68,120], while unfavorable physical conditions may reduce substrate digestibility and larval survivability. Moreover, microbiome modulation by substrate composition can indirectly affect larval health and conversion outcomes [121,122]. Therefore, the high-resolution values often cited in the literature, though statistically supported, may not reflect broader practical realities and should be contextualized within substrate-specific and environmental frameworks. Without standardization in methodology across studies, comparison of conversion efficiency outcomes remains limited, underscoring the need for more harmonized research protocols [123,124,125].

Substrate type significantly influences conversion efficiency, showcasing the superiority of BSF systems compared to traditional waste treatment methods [107,108,109,110].

### 3.2. Comparison of BSF Bioconversion and Traditional Waste Treatment Methods

BSF’s bioconversion capabilities outperform traditional organic waste management techniques such as composting, anaerobic digestion, and vermicomposting. BSF systems can reduce waste volume by up to 50%, CO_2_ processing time to 8–18 days, waste reduction by 67.91–80.39%, biomass/protein yield by 1.03–12.67% of the initial nitrogen by 0.25–4.68% and of the initial organic carbon, and land footprint (50–100 m^2^/ton of waste per day) [126,127]. Hence, producing valuable byproducts like larvae (for animal feed), frass (for fertilizers), insect oils, and chitin [15]. The BSF rapidly deodorizes organic waste through a three-fold mechanism. First, their vigorous feeding and movement physically agitate the substrate, in contrast to aerobic composting, which continues to emit terpenes, sulfur compounds, ketones, and aromatics at a rate of 2.68 × 10^7^ ou Mg^−1^ DM. In comparison, vermicomposting still releases substantial early-phase CO_2_ (130–189 g CO_2_-eq kg^−1^ DM). GHG emission estimates for BSFL systems (commonly reported as CO_2_-equivalent) typically incorporate both CO_2_, CH_4_, and N_2_O fluxes, with comparatively low CH_4_ emissions being a key advantage for climate mitigation potential. Second, larval inoculation profoundly reshapes the microbial community, sharply reducing overall diversity and depleting *Lactobacillus* and *Enterococcus*, which are key producers of volatile organic sulfur compounds like DMDS and DMTS, so that volcano-plot analyses identify 64 odorants that are diminished proportionally to larval density. Third, in direct manure trials, BSF larvae cut emissions of nine targeted VOCs (phenol, 4-methylphenol, indole, 3-methylindole, propanoic acid, 2-methylpropanoic acid, butanoic acid, 3-methylbutanoic acid, pentanoic acid) by ≥87%, achieving complete (>99%) removal of several compounds in poultry, swine, and dairy wastes. Altogether, this enables BSF processing to far outpace both composting and vermicomposting in speed, specificity, and magnitude of odor reduction [128,129,130,131,132]. Ultimately, whether the BSF “outperforms” compost or vermiculture hinges on a specific context. Under ideal BSF conditions (warm, moist, nitrogen-rich waste), the BSF can process waste faster and in a smaller area, yielding value-added larvae [133,134]. But those conditions are not universal. In cool climates or with very heterogeneous wastes, performance may drop off. Moreover, if the objective is bioenergy (biogas), conventional composting (coupled with energy recovery) might be preferable. In short, biotic factors (insect strain, gut microbes, feed type) and abiotic factors (temperature, moisture, pH, scale) critically affect outcomes. Studies emphasize that BSF results are often contextual; a “promising biotechnology” in one setting may be only marginally better (or even worse) than earthworms or composting in another [135,136]. There is no one-size-fits-all answer. BSF larvae offer high potential for rapid waste conversion on modest land area, but their advantages can be muted by practical limitations (energy capture, climate sensitivity, processing requirements) [133,134]. Conventional composting and vermiculture remain valuable, especially where BSF rearing is impractical. Researchers agree that each method’s performance is variable and situational, dependent on the waste stream and environmental conditions [136]. When comparing systems, one must consider all outputs (energy vs. protein vs. fertilizer), local climate, and infrastructure. Only then can the most suitable waste valorization approach be determined. Table 2 highlights the comparative advantages of BSF bioconversion in terms of waste reduction and environmental impact.

**Table 2:** Comparative analysis of organic waste treatment methods: BSF bioconversion versus traditional approaches.

Method	Description	Environmental Impact	Byproducts	Advantages	References
BSF Bioconversion	Using BSF larvae to convert organic waste into biomass and frass.	Reduces waste volume by up to 50% and lowers CO_2_ emissions.	Larvae (for feed), frass (for fertilizer), insect oils, and chitin.	Reduce ARGs in biosolids up to 99% in certain pathogens, minimizing heavy metal accumulation. Zinc and cobalt are not significantly retained.	[15,133]
Composting	Biological decomposition of organic matter into compost.	Reduces landfill waste, lowers methane emissions, and returns nutrients to the soil.	Nutrient-rich compost, potential liquid leachate.	Peroxydisulfate, Calcium Peroxide, and Attapulgite–Activated Carbon Composite (AACC) amendments reduce ARGs.	[137,138,139,140,141]
Anaerobic Digestion	Microbial breakdown of organic matter in the absence of oxygen, producing biogas.	It captures methane, reduces CO_2_ gas emissions, and lowers odours and pathogens.	Biogas (energy), digestate (fertilizer), and small liquid effluent.	Heavy metals and antibiotics can inhibit the activity of anaerobic microorganisms.	[142,143]
Vermicomposting	Using earthworms to convert organic waste into vermicompost.	It has a low environmental impact, reduces waste, and minimizes CO_2_ gas emissions.	Vermicompost (fertilizer), worm biomass (animal feed).	Decreases ARGs in organic waste by up to 40% in specific genes.	[137,144,145]

### 3.3. Environmental and Economic Benefits and Health Risks

BSF bioconversion offers significant environmental and economic benefits, including the production of high-value byproducts such as protein-rich larval biomass and frass and a nutrient-rich organic fertilizer that enhances soil fertility and introduces beneficial microorganisms [25,110,146]. Additionally, BSF-derived chitin is used in biopesticides, biodegradable plastics, and pharmaceuticals [25,147]. However, while the BSF offers a sustainable approach to organic waste management, challenges remain, such as potential heavy metal bioaccumulation, pathogen transmission, and allergen exposure. These risks necessitate stringent safety protocols to ensure the safety of BSF byproducts for consumption and handling [148]. Regulatory concerns regarding contaminants in feed production also need to be addressed, alongside strategies for optimizing BSF systems to maximize waste reduction efficiency and food safety [107,148,149,150]. Despite these challenges, the BSF remains a promising solution for sustainable waste management. Future research into microbial pretreatment, waste substrate optimization, and scaling up production could lead to further advancements in BSF applications for bio-manufacturing, pharmaceuticals, and renewable energy [71,107,151].

Economically, BSF rearing is considered viable. In most countries, BSF production facilities typically require an investment ranging from USD 50,762 to 100,000. However, in countries like Pakistan, the overall setup and operational costs are comparatively lower, with total facility expenses estimated between USD 10,700 and 51,100. These smaller-scale operations can still achieve profitability while supporting circular economy development and community engagement. In contrast, in high-income regions, scaling up to process approximately 110 tons of organic waste daily is often necessary to offset higher operational expenses and ensure commercial profitability [53,152,153]. The economic feasibility of converting organic waste into Black Soldier Fly (BSF) biomass for animal feed remains an area with limited research. The economic performance of BSF technology is influenced by various factors, including the location of operations, the types of substrates used, the scale of production, and the intended product application [154].

Current BSF production methods, particularly batch processes using flat tray systems, are more costly than traditional animal feed ingredients such as fish and soybean meal [155,156]. For example, a facility converting 53.6 tons of food waste into 3.64 tons of dried prepupae incurs operational costs of USD 5850 daily [156]. To achieve profitability, production costs per ton of BSF biomass should ideally not exceed USD 907 (USD 1000 per metric ton) [157]. Labor and substrate costs are the largest contributors to overall production expenses. Labor accounts for 30–65% of the total operating costs, and together, labor and substrate acquisition make up around 90% of the total cost in BSF-based animal feed production [154,158]. Substrates such as food waste, animal manure, and sewage sludge often have a negative economic value due to tipping fees for disposal [159,160]. However, government incentives, including fiscal policies for waste management, could help reduce these costs [161].

For BSF production to become profitable, production facilities must scale up, processing around 110 tons of organic waste daily to produce approximately 7.72 tons of insect meal [157]. Underused organic waste will be vital for expanding the insect-based feed industry [30]. Additionally, BSF meal, which contains lauric acid, chitin, and antimicrobial peptides, could replace conventional feed ingredients, enabling organic certification of animal and fish products, selling them at higher prices, and improving the economic viability of BSF-based feed production. Several companies, including Agriprotein (Guildford, England, UK), EnviroFlight (Apex, NC, USA), and Bioflytech (Fuente Alamo, Murcia, Spain), are engaged in BSF larvae production. A list of more companies from all over the world and their respective focus areas and products is explained in Supplementary Table S1.

Conventional composting of organic wastes typically returns nearly all of the carbon to the atmosphere: on average, it emits about 100–239 kg CO_2_-equivalent per ton of wet feedstock (roughly 100–239 g CO_2_ per kg of input) over a 120–600 day process [162]. Vermicomposting, where earthworm bioturbation maintains aerobic conditions, still produces substantial CO_2_ (130–189 g CO_2_-equivalent per kg substrate dry matter), despite suppressing methane production [162]. By contrast, BSF bio-treatment shunts approximately 50–60% of the substrate’s carbon into insect biomass, rather than into CO_2_; therefore, only 40–50% of the carbon is lost as CO_2_ [163]. Life cycle assessments of full-scale BSFL waste treatment plants find direct CO_2_ emissions of only 12–17 kg CO_2_-equivalent per ton of wet waste treated, with a further 19–73 kg CO_2_-equivalent per ton attributable to electricity for processing an order of magnitude below composting or vermicomposting [164]. When fed high-quality diets (e.g., commercial chicken mash), BSF larvae sustain high net growth efficiencies (up to 62% of assimilated carbon incorporated into biomass), minimizing respiratory CO_2_ release; on lower-quality substrates, net growth efficiency falls to around 52%, and correspondingly more carbon is respired [164]. Finally, microbial respiration within the substrate can dominate early CO_2_ emissions accounting for as much as 78% of total CO_2_ at sub-optimal moisture (2% of substrate carbon), but dropping to only 29% (3% of substrate carbon) under optimal conditions illustrating that co-occurring microbial activity critically modulates the overall CO_2_ budget of BSF bio-treatment systems [68].

#### Carbon Footprint Claims of Commercial BSF Products

The carbon footprint claims reported by insect protein companies must be interpreted with caution, particularly given the selective transparency in life cycle assessment (LCA) methodologies and the commercial interests at play. Protix (Dongen, The Netherlands) cites a carbon footprint of 0.832 kg CO_2_-eq/kg for its BSF meal ProteinX^®^, based on an LCA conducted by the DIL e.V. institute. While this figure represents substantial reductions relative to fishmeal (27%), soybean meal (71%), poultry meal (78%), and soy protein concentrate (89%), it is important to note that the underlying assumptions, system boundaries, allocation methods, and co-product handling in the LCA are not publicly disclosed. Such omissions limit external validation and raise concerns regarding methodological consistency. Similarly, InnovaFeed (Paris, France) reports a 2024 footprint of 1.6 kg CO_2_-eq/kg for its Hilucia™ Protein, a marked improvement from 7 kg in 2021 based on an LCA by Quantis (Mascot, Australlia). Although the decline is impressive, no peer-reviewed documentation has been provided, and the basis for such a dramatic reduction over a short period remains unclear. In the case of Ÿnsect’s (Genopole, France) Protein70^®^ (3.2 kg CO_2_-eq/kg) and Divaks (Vilnius, Lithuania) Textured Insect Protein (2.85 kg CO_2_-eq/kg), both based on *Tenebrio molitor*, the figures are significantly higher than for BSF-based meals, which may reflect differences in substrate use, processing intensity, or scale, but again, transparency on LCA assumptions is lacking. Moreover, Divaks’ product includes pea protein, making it difficult to attribute emissions solely to the insect component. Entobel (Singapore) and Volare (Uusimaa, Finland) have made general claims of environmental superiority but failed to provide verifiable data or third-party validation, further underscoring the opacity surrounding LCA disclosures in the insect protein sector. In sum, while insect meals, particularly BSF, show promising reductions in carbon intensity compared to conventional proteins, the heterogeneity of LCA methodologies, the lack of standardization, and the absence of peer-reviewed, publicly available data challenge the robustness and comparability of these claims. Independent validation and harmonized LCA frameworks are urgently needed to substantiate the purported climate advantages of insect-derived proteins.

These findings underscore the environmental and economic benefits of BSF bioconversion and emphasize the need for further research to optimize and scale up these systems, which is discussed in the subsequent sections. Future advancements in BSF research could lead to innovative applications in bio-manufacturing, pharmaceuticals, and bioplastics, highlighting the versatility of the BSF in various industries [71,107,165]. In summary, the BSF reduces waste volume and CO_2_ emissions and produces high-value byproducts, demonstrating clear advantages over traditional methods. The next section examines the nutritional and functional applications of the BSF in greater detail.

## 4. Nutritional and Functional Applications of BSF

### 4.1. Nutritional Composition and Feed Value

**Figure 3:**
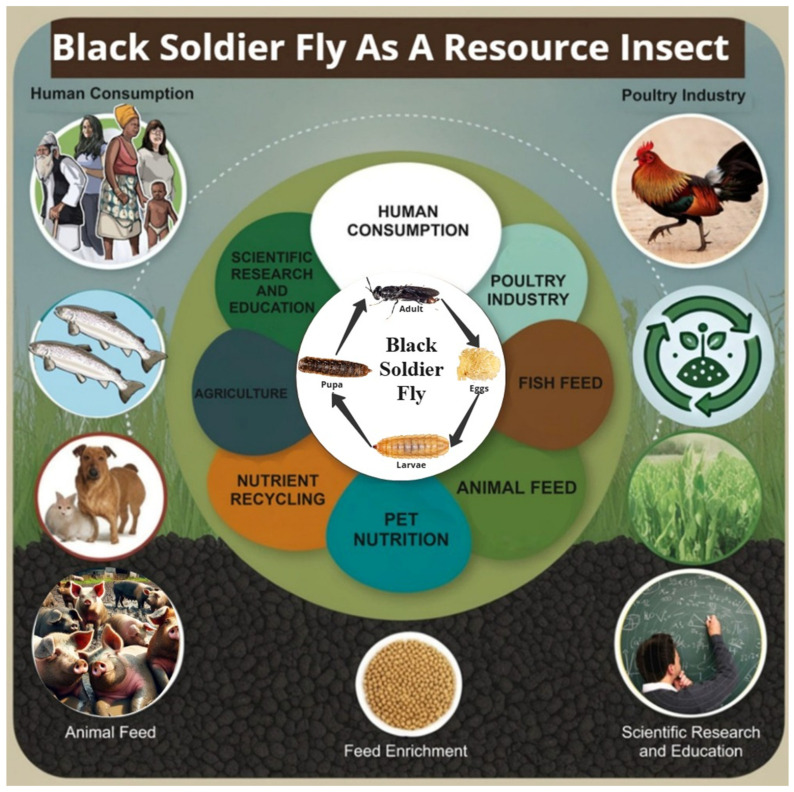
BSF as a resource insect.

The BSF has garnered significant attention as an alternative protein and lipid source due to its nutritional richness, effective bioconversion of organic waste, and broad applications in animal feed and human food industries [53,166]. To illustrate the wide spectrum of BSF’s applications as a resource insect, Figure 3 provides an overview of its roles in nutrient recovery. The nutritional composition of BSF larvae is highly dependent on substrate type, rearing conditions, developmental stage, and dietary supplementation, making substrate optimization essential to maximize their nutritional value [167,168,169].

#### 4.1.1. Macronutrient Composition

The protein content of BSF larvae generally ranges between 30% and 53%, averaging around 40%, and can reach up to 57% in defatted meals depending on the larval diet and processing methods, accompanied by a fat content of approximately 12%, predominantly consisting of saturated fatty acids, particularly lauric acid [165,170,171,172]. However, protein and fat levels vary significantly based on the rearing substrate. The quantity of proteins and lipids in BSF larvae is influenced by the feeding substrate, offering significant potential for the feed industry and enhancing the production of valuable insect-derived components. For instance, the naturally high lauric acid content in BSF larvae, which typically makes up around 50% of total lipids, can be boosted to 76% with a carbohydrate-rich diet [43,173]. Increasing the protein content in the rearing substrate raises protein levels in the larvae but also decreases fat content, highlighting the trade-off between protein and lipid accumulation [174]. This substrate-dependent modulation allows for the optimization of the larvae’s nutritional profile for various applications.

Larvae fed fish waste typically show the highest protein content, while those raised on fruit and vegetable waste exhibit the lowest [169]. Moreover, incorporating seaweed into larvae diets enriches them with omega-3 fatty acids, iodine, and vitamin E, enhancing their nutritional profile [175]. Similarly, agro-industrial byproducts can alter larvae’s crude protein, fat, and ash content, affecting growth performance and waste reduction efficiency [168,176].

The macronutrient composition also varies throughout the larval development cycle, characterized by a decrease in crude protein and an increase in crude fat as larvae mature [177]. Additionally, fermentation techniques utilizing *Bacillus subtilis WX-17*, *Aspergillus niger*, and *Bacillus coagulans* can significantly enhance nutritional attributes of substrates, including increases in amino acids, unsaturated fatty acids, and crude protein content [178,179]. Carbohydrate composition similarly demonstrates substrate-dependent variability, influencing the larvae’s overall nutritional and structural profile [172,174]. Larval lipid profiles predominantly comprise triacylglycerol-bound fatty acids, with limited opportunities for dietary modifications to significantly alter their saturation, unsaturation, or methyl branching [180].

#### 4.1.2. Micronutrients

BSF larvae contain important micronutrients, including vitamins and trace minerals critical for animal nutrition. Essential vitamins identified in BSF larvae include vitamin B1 (thiamine), B2 (riboflavin), and vitamin C, all of which contribute to their nutritional value [181]. Additionally, strategies such as gut-loading larvae with dietary vitamin A at concentrations of 16,000–20,000 mcg retinol equivalents/kg for 24 h at optimal moisture (around 60%) and density conditions (0.1–1 larvae per gram of substrate) effectively enrich the larval micronutrient profile [182]. These micronutrients collectively augment the larvae’s health-promoting properties, positioning BSF larvae as a valuable nutritional resource suitable for diverse applications in animal and human nutrition sectors.

#### 4.1.3. Mineral Content

BSF larvae are notably rich in essential minerals, including potassium (K), calcium (Ca), phosphorus (P), iron (Fe), manganese (Mn), magnesium (Mg), sodium (Na), zinc (Zn), and copper (Cu), all of which vary according to substrate type and processing methods [181]. Substrate-driven modulation enables recovery of key macronutrients from otherwise discarded biomass, underpinning circular nutrient flows. For instance, substrates like banana peels have been shown to significantly enhance the mineral content. By choosing a substrate blend (e.g., banana peels + other wastes), you can “program” your larvae to accumulate a tailored mineral profile [183]. Through this process, BSFL not only valorize organic residues into mineral-rich biomass for use as a sustainable feed ingredient but also generate frass with fertilizer-grade mineral content, supporting soil health while mitigating landfill burdens and eutrophication risks [25]. Processing methods such as spray-drying and oven-drying also influence the final mineral concentration, highlighting the importance of handling methods to preserve nutritional integrity [181].

#### 4.1.4. Bioactive Compounds

BSF larvae contain a variety of bioactive compounds, including phenolic substances (hydroxybenzoic and hydroxycinnamic acids, flavonoids, anthocyanins, ellagitannins), essential vitamins (B1, B2, C), and beneficial polyunsaturated fatty acids (PUFAs) like conjugated linoleic acid (CLA), docosahexaenoic acid (DHA), and eicosapentaenoic acid (EPA) [184,185,186,187,188]. These compounds confer antioxidant and anti-inflammatory properties, significantly enhancing the larvae’s potential as functional food ingredients. Encapsulating these bioactives through micro- or nano-encapsulation techniques can further improve their stability and bioavailability [189].

**Figure 4:**
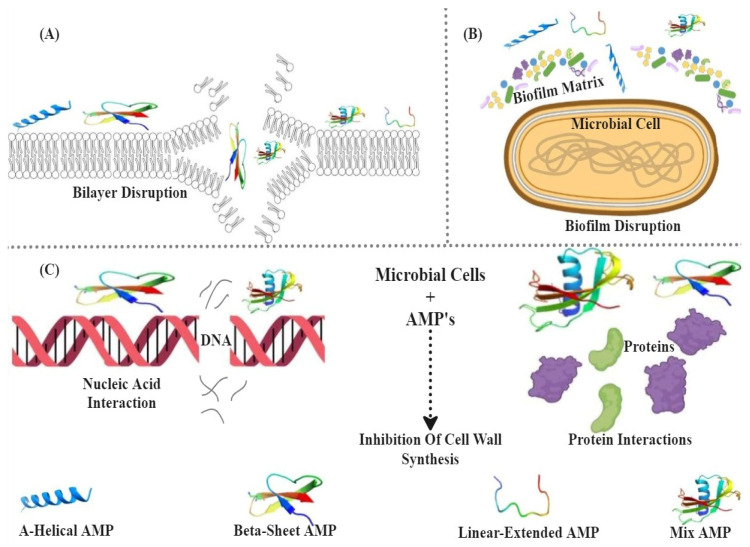
Mechanism of action of AMPs: (**A**) lipid bilayer disruption of microbial cells by AMPs, (**B**) destruction of microbial biofilm layer by AMPs, (**C**) interaction of AMPs with DNA and inhibition of cell wall synthesis by AMPs, and interaction of AMPs with proteins.

BSF larvae are rich in antimicrobial peptides (AMPs) such as cecropin-like peptides, defensins, and diptericins, exhibiting strong antimicrobial and anti-inflammatory properties by targeting various pathogens (Figure 4 illustrates primary antimicrobial mechanisms of peptides) through membrane disruption and inhibition of biofilm formation [71,190,191]. Larval extracts containing lauric acid also demonstrate antimicrobial efficacy against pathogens like *E. coli* and multidrug-resistant bacteria, making them valuable for medicinal and agricultural applications [192]. Enzymes such as proteases, lipases, and chitinases produced by BSF larvae are essential for effective waste degradation and nutrient absorption, with applications extending to pharmaceuticals, biopesticides, plant growth enhancement, cosmetics, and sustainable animal feed production [110,193,194,195,196].

#### 4.1.5. Comparative Context

Black Soldier Fly larvae (BSFL) meal is high in protein (30–53% reaching 57% if defatted), similar to or somewhat below premium fish meals (60–72% protein) and generally higher than soybean meal (45–53%) [197,198]. BSFL also contains very high lipid (15–40%), far more than soybean meal (only a few percent), and comparable (see Table 3 and Table 4 for a comparison) to fish meal (roughly 6–10%) [197,198,199]. Conversely, BSFL is relatively rich in indigestible chitin (often 6–9% fiber) and ash (6–8%), reflecting its mineralized exoskeleton, whereas soybean meal has moderate fiber and ash (6–7%), and fish meal is virtually fiber-free but very ash-rich (18–25%) [197,198,199,200]. Consequently, BSFL meal delivers substantial calcium (from chitin/cuticle, often on the order of tens of g/kg) and moderate phosphorus levels much higher than in plant meals (soybean Ca 3–5 g/kg) and roughly in line with or exceeding fish meal (which is Ca-rich due to bone). In amino acids, BSFL provides a well-balanced profile. Its essential amino acid pattern closely resembles fish meal and typically exceeds soybean meal in the limiting Lys and Met, whereas soybean meal, although relatively high in total protein, is lower in these amino acids (and contains anti-nutritional factors) [197]. Finally, fatty acid profiles differ sharply: BSFL fats are dominated by saturated chains, especially lauric acid (C12:0 ~20% of BSFL fatty acids) and moderate MUFA, with low n-3 PUFA content [199]. By contrast, soybean meal’s residual fat (and added plant oils) is rich in C18:1 and C18:2 (n-6), and fish meal provides significant long-chain n-3 PUFAs (EPA, DHA) not found in BSFL. Taken together, BSFL meal offers protein and amino acid quality approaching that of fish meal (and generally superior to soybean meal), with much higher fat (largely saturated) and mineral content (high Ca, ash) than plant meals; soybean meal is high-protein but low-fat and low-mineral; and fish meal is very protein-rich with moderate fat (including omega-3 PUFA) but high ash [197,199]. BSFL systems support circular nutrient flows by recycling food and agricultural residues into high-value protein and minerals, reducing reliance on soybean cultivation and overfished stocks [201]. Nevertheless, substrate-driven variability in BSFL nutrient composition and potential heavy metal bioaccumulation underscore the need for routine safety assessments to match the consistency of SBM and FM.

**Table 3:** Comparative nutritional composition of Black Soldier Fly larvae (BSFL), soybean meal (SBM), and fish meal (FM).

Nutrient	BSFL	SBM	FM
Crude Protein%	30–53 (dry matter)	41.39	56
Crude Fat%	20–41	1.18	8.6
Crude Fiber%	2–9 (due to chitin)	0.87	0
Ash%	2–9	6.3	15
Calcium (Ca)%	3.85	0.29	7.38
Phosphorus (P)%	0.94	0.56	3.97
Sodium Chloride (NaCl)%	0.36	0.06	0.7
Amino Acids%	Comparable to FM; higher than SBM	Lower levels	Higher levels
Fatty Acids%	Lauric (21%), Oleic (32%), Palmitic (16%).	Lauric (trace), Oleic (18%), Palmitic (11%).	Lauric trace, Oleic (19.8–27.1%), Palmitic (21.2–26.6%).

References: [40,54,181,202,203,204,205,206,207,208].

**Table 4:** Nutritional composition comparison of resource insects.

Insect	Protein (%)	Carbohydrates (%)	Fats (%)	Ash (%)	Micronutrients (%)	Chitin (%)	Reference
Crickets (*Acheta domesticus*)	65	21	6	2	3	3	[209]
Mealworms (*Tenebrio molitor*)	50	38	13	1	3	1	[210,211,212]
Grasshoppers (*Caelifera*)	70	15	5	3	2	2	[213,214,215]
Silkworms (*Bombyx mori*)	64	21	10	4	1	2	[216,217,218]
Black Soldier Fly (*Hermetia illucens*)	42	30	35	3	2	1	[54]
Ants (*Formicidae*)	42	25	16	2	4	1	[219]
Termites (*Isoptera*)	35	23	28	3	4	3	[220,221]
Locusts (*Locusta migratoria*)	60	20	10	5	2	3	[215,222]

In summary, the multifaceted nutritional composition of BSF larvae, influenced by diet, developmental stage, and processing techniques, positions them as a versatile and sustainable alternative for nutritional and functional applications across various industries.

## 5. Industrial Applications of the BSF

**Figure 5:**
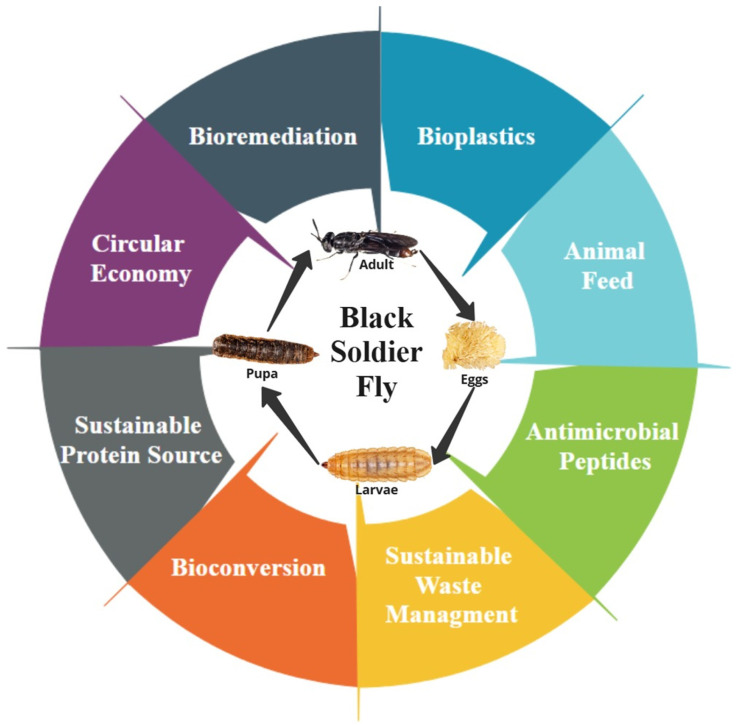
Applications of the BSF.

While we have detailed BSF’s genetic and physiological adaptations, the following explores how these adaptations translate into industrial applications, including animal feed, waste management, bioremediation, bioplastics, and circular economy integration. Figure 5 provides a comprehensive overview of BSF’s diverse industrial applications, illustrating how organic waste is converted into high-protein biomass, biofuels, and bioplastics.

### 5.1. Animal Feed

The BSF demonstrates its potential as a sustainable, high-protein feed source for livestock and aquaculture. The BSF can convert plant-based organic waste into valuable biomass, making it a viable alternative to traditional protein sources in chicken and fish diets, enhancing growth and carcass quality [223,224,225,226,227]. Environmental factors such as moisture levels, pH, and feeding systems are critical for optimal larval growth, with 70% moisture and daily feeding regimens being particularly effective [228,229]. The BSF has further potential in immunomodulation, enhancing immune responses in broiler chicks and serving as a protein replacement in salmonid meals [230,231,232]. However, heavy metal contamination in substrates poses a risk, highlighting the need for careful monitoring [228]. The BSF and mealworm used as environmental enrichment in broiler diets show no negative impact on growth or health. The BSF has positively influenced African catfish growth and body index in aquaculture, although substrate safety remains a concern [228,233]. Understanding the regulatory landscape is critical for the commercialization of insect-based feeds.

The BSF is a valuable alternative protein source for livestock, poultry, and aquaculture, offering high protein content that supports sustainable feeding without compromising forage utilization [234,235]. Their production aligns with circular agriculture by recycling minerals from waste streams and efficiently converting organic waste into valuable biomass, providing significant economic and environmental benefits [85,236,237]. As shown in Table 4, although the BSF exhibits a relatively lower protein content (42%) than grasshoppers and crickets, it offers a higher fat content, which may be advantageous for certain feed applications. BSF’s rich nutritional profile can replace meat bone meal in chicken diets, improving feed conversion ratios, though regulatory challenges remain for broader adoption [71,107,151]. The trade-off in Table 4 suggests that processing techniques to improve digestibility may be necessary to maximize the feed value of the BSF. For instance, the higher fat content in the BSF might be optimized through specific processing techniques and substrate modification to enhance digestibility and energy yield, thereby supporting its application in aquaculture and livestock diets. In summary, although regulatory and substrate safety challenges persist, the BSF demonstrates considerable potential as an alternative protein source for animal feed.

### 5.2. Sustainable Waste Management

The BSF plays a key role in sustainable waste management by converting organic waste, reducing landfill use, and producing frass, a nutrient-rich fertilizer that boosts plant growth [53,238,239]. In pharmaceuticals, BSF compounds aid in waste reduction and influence amino acid content in animal products, with the BSF reducing biosolids, pathogens, and ARGs, offering the potential for new antimicrobial treatments [47,53,152]. BSF biomass is also used in biofuel production, with processes like catalytic fast pyrolysis (CFP) over zeolites enhancing bio-oil quality and agricultural residues serving as feedstock, supported by catalysts that increase yield and reduce CO_2_ emissions [240,241]. The BSF contributes to ecological stewardship by minimizing landfill waste and reducing methane emissions associated with organic waste decomposition [53]. As shown in Table 2, the comparative analysis reveals that BSF bioconversion significantly reduces waste volume while producing valuable byproducts, lowering CO_2_, and outperforming conventional methods (see Section 3.3). The BSF’s ability to thrive on various organic substrates underlines its adaptability and effectiveness in diverse environmental conditions. These applications underscore BSF’s crucial role in promoting sustainability across multiple sectors. In summary, BSF-based systems effectively reduce organic waste and CO_2_ emissions while producing valuable byproducts.

### 5.3. Bioremediation and Soil Enhancement

The BSF demonstrates significant potential in bioremediation and soil enhancement. This process is facilitated through the BSF’s unique ability to recruit functional microbiota, enhancing the degradation of complex organic materials. The BSF significantly improves the degradation of lignocellulosic waste, achieving a biodegradation rate of 26.5% compared to only 4.06% in natural composting [88]. The BSF’s intestines harbor a diverse community of lignocellulose-degrading bacteria, crucial for efficient waste processing [88]. Pathogen and antibiotic resistance reduction BSF bioconversion of biosolids has been shown to reduce pathogenic bacteria and antibiotic resistance genes by over 99%, making it a promising technology for waste management [47]. Although early work identified a nutrient profile for BSF frass of roughly 5:2:2 (N:P:K), a meta-analysis of multiple studies suggests that these proportions are neither fixed nor universally applicable [242,243]. In fact, aggregated data indicate an average frass composition closer to 10:9:11, but with marked variability: nitrogen content across commercial products spans 1.1–8.0%, phosphorus 1.0–8%, and potassium 1–17%. The comparatively low P and K levels often reported reflect the inherent challenges in recovering these elements from complex insect-rearing substrates [24]. Moreover, another study demonstrated that BSF frass can contain up to 130% more nitrogen and 193% more potassium than frass from eight alternative insect species; these enhancements underscore both the promise and the inconsistency of BSF-derived fertilizers [244]. Taken together, the data argue for standardized substrate protocols and robust quality-control measures if BSF frass is to reliably substitute for conventional NPK fertilizers. The BSF can recycle nitrogen from biowaste, converting it into protein-rich biomass while minimizing nitrogen losses, thus addressing environmental issues like eutrophication [245]. The BSF efficiently degrades organic pollutants and bioaccumulates heavy metals like cadmium and lead, making them valuable for cleaning contaminated sites and converting hazardous waste into protein- and fat-rich biomass (to use the BSF in feed, one must ensure substrate safety, apply post-harvest detoxification, and rigorously monitor metal levels) [246,247,248]. The frass produced by the BSF is recognized as an effective soil amendment, rich in essential nutrients like nitrogen, phosphorus, potassium (NPK), and chitin, which improves soil structure, fertility, and plant immunity [27,28,249]. The BSF exhibits promising bioremediation capabilities by degrading pollutants and improving soil quality.

### 5.4. Bioplastics

In the bioplastics industry, BSF chitin is processed into chitosan, a versatile biopolymer used in biodegradable films, packaging, and medical applications, while BSF-derived proteins enhance bioplastic formulations, offering a sustainable alternative to conventional plastics [250,251,252]. BSF-derived products also find applications in cosmetics, where AMPs and chitosan are used for their antibacterial, skin-healing, and anti-aging properties, and in cosmetic and food storage, where the BSF provides a high-protein, environmentally friendly alternative to traditional proteins [250,253]. Furthermore, the BSF is explored for producing industrial enzymes, contributing to waste treatment, and biofuel production. These applications underscore BSF’s versatility and role in promoting sustainability across multiple sectors. Major research using the BSF to generate protein-based bioplastics found that films formed from soluble protein fractions had good tensile qualities, transparency, and water resistance, particularly when moderate crosslinking agents like citric acid are applied. Bioplastics’ red light resilience indicates agricultural applications such as biodegradable pots, mulching films, utensils, and food and non-food packaging. BSF-derived bioplastics may minimize environmental impact by replacing traditional plastics with biodegradable ones, especially in businesses that demand sustainable packaging [254]. BSF-derived chitin, primarily harvested from prepupal exuviae at purities up to 96%, presents a viable alternative to crustacean sources for bioplastic production, given its comparable crystallinity and mechanical performance, tensile strengths of 38–85 MPa, and water vapor transmission rates of 5–10 g·m^−2^·d^−1^ when formulated into chitosan films [255,256]. Adoption of green extraction protocols, such as glycerol–HCl co-solvent systems and enzymatic or mechano-chemical deproteination, can minimize chemical waste and yield chitosan with degrees of deacetylation exceeding 80%, thereby imparting antimicrobial functionality and robust film-forming capacity [256,257]. Incorporation of smectite nanoclays (sepiolite or montmorillonite) into insect-derived chitosan matrices enhances UV resistance and mechanical reinforcement, though reliance on synthetic crosslinkers raises valid concerns over potential residual toxicity in food-contact applications [257]. While Fourier-transform infrared spectroscopy and X-ray diffraction analyses confirm that BSF-chitin mirrors the structural integrity of marine-derived polymers, variability in insect diet and farm conditions can introduce heterogeneity in polymer molecular weight and crystallinity, complicating efforts toward industrial standardization [255,258,259]. Techno-economic assessments suggest that leveraging existing BSF farming infrastructure for chitin extraction could markedly reduce reliance on declining marine chitin stocks and support circular bioeconomy models, yet pilot-scale cost–benefit data and supply-chain integration studies remain critically lacking [260,261]. Emerging applications in biodegradable packaging, agricultural mulch films, and 3D-printed biomedical scaffolds underscore the versatile potential of insect-derived chitin, but the absence of insect-specific regulatory guidelines for biopolymer safety and quality constitutes a significant hurdle to commercialization [260,262]. Advances in mechano-chemical pretreatment strategies have further demonstrated chitin purities exceeding 95% while reducing acid and alkali usage, bolstering the environmental credentials of BSF-chitin-based bioplastics [263]. BSF chitin and proteins offer viable, sustainable alternatives to conventional plastics.

### 5.5. Circular Economy

**Figure 6:**
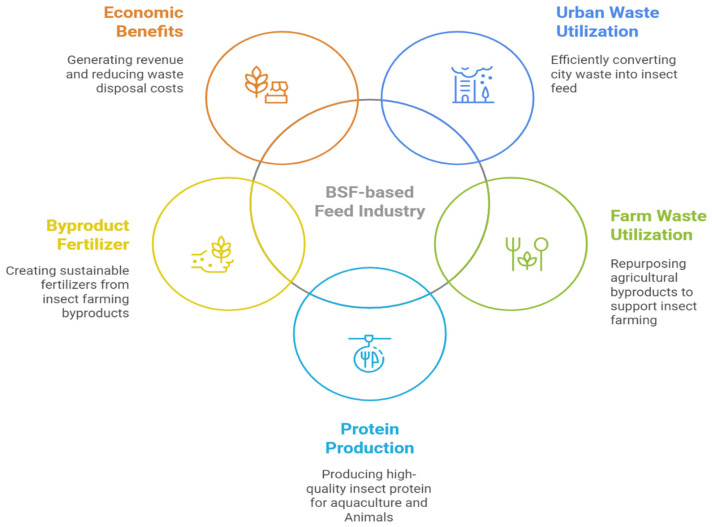
BSF-based feed industry integration into the circular economy.

The BSF contributes to circular economy models and sustainability by efficiently converting organic waste into valuable resources such as high-protein biomass and nutrient-rich frass, which can be used as animal feed and organic fertilizer [96,251]. This process reduces the waste sent to landfills and recycles nutrients back into the ecosystem, supporting sustainable agricultural practices. Integrating BSF into waste management systems reduces reliance on synthetic inputs and lowers environmental impacts, aligning with sustainability goals and minimizing CO_2_ emissions (see Section 3.3) [250,264]. The integration of BSF-based feed into circular economy frameworks is graphically depicted in Figure 6, illustrating the flow of waste conversion, resource recovery, and nutrient recycling within sustainable agricultural systems. Recent research shows that the BSF uses waste reduction and resource recovery to make animal feed, lipids, chitin, and fertilizer (frass), suggesting a circular economy. A circular bio-economy may be promoted by turning low-value byproducts into protein meal and oil. IoT-enabled BSF farming systems have proven capable of transforming over 80% of diverse organic wastes into larval biomass and frass within two weeks by continuously monitoring and automating key parameters, temperature, humidity, pH, and aeration using platforms from ESP8266 sensor networks to commercial “smart bins” [265,266]. Yet their circular economy promise is tempered by persistent hurdles: sensor drift and connectivity gaps in off-grid or rural settings undermine data reliability, while the upfront and maintenance costs of heterogeneous IoT architectures (ESP8266, WaspMote, SenseCAP, FlyFarmOS) impede adoption among smallholders [266,267,268]. Moreover, few studies quantify the IoT footprint energy for device fabrication, data transmission, and cloud computing, which could offset environmental gains unless addressed through energy-efficient edge computing and comprehensive life cycle assessments [269]. Moving forward, standardizing open protocols for device interoperability, integrating blockchain for substrate-to-product traceability, and leveraging machine learning on IoT time-series data will be critical to realize truly resilient, scalable BSF operations within circular economy frameworks. They can also produce chitin from insect biowaste, which has applications in cosmetics and bioplastics [270]. Economic opportunities in the BSF industry are rapidly commercializing, providing a sustainable alternative to traditional livestock feed, thus supporting food security and resource conservation [53]. This innovative approach addresses waste management challenges and contributes to sustainable food production systems. Thus, BSF integration into circular economy frameworks effectively recycles organic waste into valuable biomass.

### 5.6. Bioconversion

**Figure 7:**
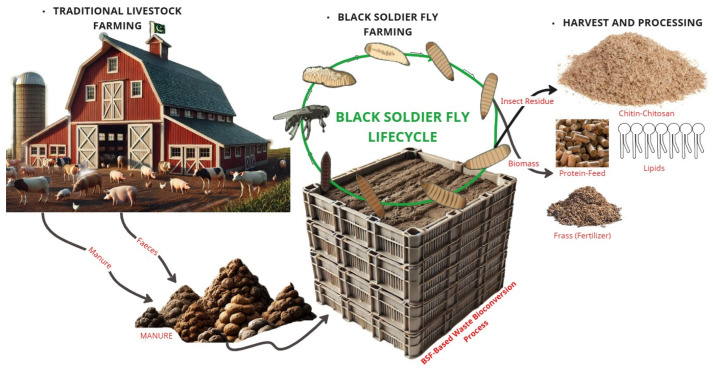
BSF-based bioconversion of livestock waste.

The bioconversion of organic waste using the BSF is highly promising, with the BSF efficiently converting waste like fermented maize straw into protein- and fat-rich biomass [271,272,273]. The BSF has been successfully used in food waste treatment and energy generation, especially in Asia, and shows potential for biofuel and animal feed production [274,275]. Additionally, the BSF offers significant agricultural benefits by providing a low-cost protein source for poultry and fish production and contributing to sustainable waste management by converting organic waste into animal feed [223,276]. Their ability to reduce municipal, food, and livestock waste while yielding high-protein, lipid-rich biomass, and balanced NPK frass underscores their role in waste conversion [13,277,278]. The BSF bio-converts substrates into usable products for sustainable waste management. Research shows that the BSF can convert mushroom substrates, wet distiller’s grains, kitchen trash, manure, and biosolids into protein- and fat-rich biomass. BSFL can convert SMS (spent mushroom substrate), WDG (wet distiller’s grains), and their mixtures; 100% WDG is suitable for the growth of the BSFL and can promote the humification of the substrate during the treatment process, increasing the content of total kjeldahl nitrogen, total organic carbon, total phosphorus, and total potassium in the residues [279]. Another study found that the BSF produces feed-quality biomass and reduces kitchen and fecal waste by 70% [52]. Optimization of substrate particle size reduced waste, and larval growth increased bioconversion [280]. A good hazardous waste management method, biosolids bioconversion, decreased dangerous bacteria and ARGs [47,279,281,282,283]. These findings imply that the BSF might be a promising bio-converter of resources from organic waste. Figure 7 illustrates a BSF-based waste bioconversion system. In summary, the BSF demonstrates high bioconversion efficiency by transforming diverse waste streams into nutrient-rich biomass.

### 5.7. Sustainable Protein Source

The BSF is a nutritionally dense and sustainable protein source, with protein content ranging from 38% to 60%, depending on their developmental stage, and fat content up to 28.4%, including essential fatty acids like omega-3 [168,175,177,183]. The BSF nutritional profile, including vital micronutrients such as vitamin E, iodine, calcium, and phosphorus, can be modulated by their diet, making them versatile for animal feed and potential human consumption [167,175,284]. The BSF is comparable to fishmeal and superior to soy in essential amino acids like lysine and methionine, and they offer additional health benefits through lauric acid, which has antimicrobial properties [284]. Studies have shown that the BSF can improve animal growth rates, feed conversion ratios, and immune responses, making them a highly nutritious and environmentally friendly feed option compared to conventional fishmeal and soybean-based feeds. In a 42-day feeding trial, replacing 15–20% of conventional broiler feed with full-fat BSFL whose biomass contains 40% crude protein, 32% fat (notably lauric acid), balanced amino acids, chitin, and antimicrobial peptides [285,286]. BSF bioactive components have been found to play a significant role in reducing both peripheral (36–48%) and intestinal intraepithelial CD3+ and CD8+ T-lymphocyte populations up to ten-fold. This reassuring finding underscores the potential of these components in managing immune energy costs [287]. Processing methods like drying, grinding, and fermenting further enhance the nutritional value of BSF, increasing the bioavailability of essential nutrients and reducing microbial loads, solidifying their role as a sustainable alternative to traditional protein sources [288,289,290]. Recent research reveals that the BSF might recycle organic waste into protein- and lipid-rich biomass for cattle feed. In a multitrophic growing system, the BSF turns kitchen waste into high-protein biomass for animal or aquaculture feed [291]. The BSF turns animal droppings into protein and organic fertilizers, minimizing waste and environmental issues [292]. Further trials show the BSF may transform waste into products and minimize protein usage, validating circular bio-economy models [53]. Legal and public approval issues impede BSF-derived animal feed product commercialization [55]. Studies show that the BSF may improve food security and the environment as a sustainable protein source. The BSF offers a robust nutritional profile, positioning it as a sustainable alternative to conventional protein sources.

### 5.8. Antimicrobial Peptides (AMPs) and Species-Specific Applications

The BSF is a rich source of AMPs that play a vital role in their innate immune system, showing promising antimicrobial activity against various pathogens [293]. Notable AMPs such as *Jg7197.t1*, *Jg7902.t1*, and *Jg7904.t1* have been identified and predicted to combat bacterial strains like *Pseudomonas aeruginosa* [293]. These AMPs inhibit microbial growth by disrupting cell membranes, interfering with DNA replication, and preventing biofilm formation, making them effective against bacteria, fungi, and parasites [294,295,296,297]. Specific AMPs, such as cecropins and defensins, offer promising applications in medical fields, including antibacterial therapies and wound care [93,298,299]. For a detailed overview of the antimicrobial mechanisms, effective targets, and applications of these peptides, refer to Table 5. Moreover, these AMPs hold potential as natural pesticides and feed additives in agriculture, providing an eco-friendly alternative to chemical treatments and enhancing soil health [294,295,300]. Beyond antimicrobial and anti-inflammatory benefits, BSF-derived peptides also offer potential antioxidant and immune-modulating properties, making them valuable for functional foods and nutraceuticals to enhance human health [301,302,303]. Figure 4 illustrates the three primary antimicrobial mechanisms of BSF-derived peptides: (A) disruption of the lipid bilayer, (B) degradation of biofilms, and (C) interference with DNA and protein synthesis, each contributing to their broad-spectrum activity.

However, commercializing BSF-derived AMPs faces challenges such as high production costs, regulatory hurdles, and competition with synthetic alternatives [296,304]. Despite these challenges, the rising demand for natural, sustainable products and new solutions in the face of antibiotic resistance presents significant opportunities for AMP-based products in healthcare, agriculture, and environmental sustainability [295,305]. Strategic partnerships could help overcome these barriers, allowing AMPs to capture emerging markets [306]. In conclusion, BSF-derived antimicrobial peptides exhibit significant promise as natural alternatives to synthetic antimicrobials, with potential applications across healthcare and agriculture.

**Table 5:** BSF-derived antimicrobial peptides’ mechanism of action, effective against, and their uses.

Type of AMP	Mechanism of Action	Effective Against	Uses	References
Defensins	Disrupt bacterial cell membranes, broad-spectrum activity	Effective against Gram-positive bacteria such as *Staphylococcus aureus* and Gram-negative bacteria such as *E. coli*	Potential use in agriculture and medicine as natural antimicrobial agents	[307,308,309]
Cecropins	Disrupt bacterial cell membranes	Effective against a broad range of bacteria, including *E. coli* and *S. aureus*	Useful as natural antimicrobial agents in agriculture and medicine	[310,311,312]
Diptericins	Target Gram-negative bacteria by binding to cell wall components	Highly effective against Gram-negative bacteria such as *Pseudomonas* spp.	Potential use in agriculture and medicine as natural antimicrobial agents	[307,311,313]
Attacins	Target the bacterial cell envelope, disrupt cell wall synthesis	Effective against Gram-negative bacteria, including *E. coli* and *Klebsiella* spp.	Potential use in agriculture and medicine as natural antimicrobial agents	[293,314,315]
Proline-Rich Peptides	Penetrates bacterial cell walls, inhibits intracellular targets such as protein synthesis	Effective against Gram-negative bacteria and certain Gram-positive bacteria	Useful as natural antimicrobial agents in agriculture and medicine	[295,309,316]
Lysozyme	Hydrolyze peptidoglycan layer in bacterial cell walls	Highly effective against Gram-positive bacteria like *Staphylococcus aureus* and *Streptococcus* spp.	Useful as natural antimicrobial agents in agriculture and medicine	[295,317,318]
Moricin-Like Peptides	Disrupt bacterial cell membranes	Effective against a wide range of bacteria, including *E. coli* and certain viral pathogens	Useful as natural antimicrobial agents in agriculture and medicine	[297,310,319]
Attacin-Like Peptides	Target bacterial cell envelope, disrupt cell wall synthesis	Effective against Gram-negative bacteria, including *Pseudomonas* spp. and *E. coli*	Potential use in agriculture and medicine as natural antimicrobial agents	[309,314,320]
Other AMPs	Target bacterial cell membranes	Effective against a variety of bacterial species, including multidrug-resistant strains	Useful as natural antimicrobial agents in agriculture and medicine	[309,314]

## 6. Challenges and Prospects

Industrial-scale deployment of *Hermetia illucens* processing is hampered above all by a fragmented and risk-averse regulatory landscape. In the European Union (EU), Regulation 2021/1372 has lifted some of the BSE-era bans on insect proteins. However, it still restricts certain high-value waste streams, such as catering refuse and manure. In the United States, insect meal is permitted only in aqua feeds for salmon, according to the rules set by the Association of American Feed Control Officials (AAFCO). In Canada, the approval of insect ingredients is granted on a case-by-case basis. [321]. Such patchwork governance forces producers into costly, duplicative testing regimes (heavy metals, pesticide residues, microbial pathogens) without insect-specific guidance, and perpetuates uncertainty around acceptable substrates and end-uses [322].

Technical and market challenges compound regulatory obstacles. Scaling larvae production demands tight control of substrate quality and environmental parameters to ensure consistent yields of protein, oil, chitin, and AMPs, yet waste streams vary widely in nutrient composition and contaminant load. Bioconversion bioreactors must balance throughput with hygienic processing to prevent spoilage or pathogen proliferation, increasing capital and operating expenses. Meanwhile, immature supply chains and limited consumer awareness constrain downstream markets for insect-derived ingredients and frass fertilizers. Industry associations such as IPIFF (International Platform of Insects for Food and Feed) are urging harmonized, insect-specific safety standards, defining clear contaminant thresholds and welfare criteria to unlock substrate flexibility, reduce compliance overhead, and build investor confidence [322].

A coherent global framework combining explicit hazard limits, substrate approvals, and streamlined authorization pathways is urgently needed to bridge divergent regulations, de-risk investments in scalable infrastructure, and catalyze market uptake of BSF bioproducts. Emotion-neutral, evidence-based policy reform will be pivotal to transform promising lab-scale processes into commercially viable, circular solutions for organic waste valorization.

### 6.1. Regulatory Landscape and Policy Challenges

Looking beyond the bans on catering waste, manure and feed only AAFCO approvals, the EU’s approach to insect-derived foods, including BSF larvae and powders, as novel foods under Regulation 2015/2283, is thorough. This involves the creation of exhaustive EFSA (European Food Safety Authority) dossiers, allergenicity testing, and safety opinions. While only four species have been authorized to date and BSF applications remain under review, this process, though it may cause delays, ultimately ensures the safety and quality of insect-derived foods, delaying market entry by 2–5 years. The potential benefits of this process are significant, promising a future of safe and high-quality insect-derived foods [321,323]. The United States has no insect-specific food law; instead, it subsumes edible insects under the Federal Food, Drug, and Cosmetic Act and generic FSMA (Food Safety Modernization Act) preventive controls, treating insect parts as potential adulterants, and lacking a clear regulatory pathway for BSF human foods [324,325]. Canada’s Division 28 novel food framework under the Food and Drugs Act and Safe Food for Canadians Regulations requires comprehensive compositional, chemical, microbiological, and toxicological data, plus a 60-day novelty determination for every new insect species, yet no BSF ingredient has completed this process [326]. Singapore’s Food Agency mandates that BSF larvae undergo its novel foods approval process, complete with formal risk assessments and safety documentation, before import or sale for human consumption [327]. In China, edible insects, including BSF, fall under the general Food Safety Law without species-specific standards for rearing substrates, contaminant limits, or processing methods, creating regulatory uncertainty that hinders the domestic human food market for BSF proteins [328,329].

### 6.2. Feedstock and Process Engineering

Industrial-scale BSFL processing is fundamentally constrained by feedstock heterogeneity, which drives significant variability in larval growth and biochemical composition: high-fiber or protein-deficient substrates depress larval protein and lipid yields, undermining standardization of insect meal and oil [55]. Moreover, BSFL bioaccumulate heavy metals, sequestering 70–90% of Zn, Cr, and Cu from contaminated feeds, resulting in frass with lower metal loads but persistent risks of mycotoxins, dioxins, pesticides, veterinary drugs, and pathogens such as *Salmonella* and *Bacillus cereus* [330]. Raw frass is also moisture- and ammonia-rich, necessitating thermophilic composting or passive drying to mature the material, mitigate phytotoxicity, and stabilize microbiological quality [55]. To safeguard circularity, operators must implement robust quality-control regimes, substrate blending, sorting, pasteurization, and continuous contaminant monitoring at each stage [55,330].

On the engineering front, reliance on manual climate control (27–30 °C; 60–70% RH) yields inconsistent rearing conditions, while mechanical separation via two-stage vibratory sieves achieves only 2–3% frass purity at the expense of 4–6% larval injury [331]. Energy-intensive hexane/ethanol extraction for lipids and proteins remains the norm, despite emerging greener alternatives (subcritical water, enzymatic, ultrasonic) that have yet to reach commercial maturity. Stakeholders advocate automation, IoT-enabled chambers, automatic feed mixers, vision-guided harvesters, and modular, open-source rearing units to reduce labor costs, stabilize production, and lower capital barriers [51].

### 6.3. Product Quality, Safety, and the Consumer

Ensuring consistent product quality and safety is essential for market uptake of BSFL-derived ingredients. Although regulators universally mandate heavy metal, pesticide, and microbial screening, insect-specific threshold values are largely absent, despite metal accumulation in larvae varying by over an order of magnitude with diet [321,322]. Allergenic risks from chitin and tropomyosins can be attenuated via defatting, chitin fractionation, or enzymatic hydrolysis, while the inherent production of antimicrobial peptides (defensins, cecropins) offers a valuable co-product, albeit one with low native yields and heat/pH-sensitivity that demands breeding or dietary modulation alongside advanced encapsulation or stabilization approaches for viable recovery [36]. Regulatory pathways for insect-derived AMPs remain ambiguous, oscillating between feed-additive and pharmaceutical classifications, so proactive engagement with authorities on safety and efficacy testing is advisable.

In Western consumer markets, persistent neophobia toward whole insects extends even to processed formats such as flours and bars, slowing adoption rates [44]. Education campaigns, sensory trials, and framing insect proteins in familiar product formats, coupled with transparent sustainability credentials, may incrementally shift perceptions. Concurrently, ethical debates are emerging around BSFL welfare; although larvae are natural saprophages, concerns over high-density rearing and waste-based substrates call for consideration of optimal stocking densities, humane harvesting methods, and industry codes of practice to build consumer trust [44,332]. Clear labeling, stakeholder engagement, and development of insect-specific safety and welfare guidelines will be pivotal to secure broad acceptance of BSFL bio-products.

### 6.4. Environment and Economy

Life cycle assessments consistently show that BSFL production can outperform conventional livestock in greenhouse gas intensity, often by two to three orders of magnitude per kilogram of protein, when larvae are reared on low-energy wastes, but these benefits are highly contingent on energy sourcing and process design [44,164]. Electricity-intensive operations, climate control, feedstock preprocessing (e.g., freezing, chopping), and frass drying can erode, or even reverse, the climate advantage if powered by fossil-based grids (See Table 6 for key challenges). Co-locating BSF facilities with anaerobic digesters or other waste treatment plants offers methane capture and heat integration, while passive architectural design and on-site solar generation can reduce grid reliance [44]. In terms of environmental sustainability, life cycle assessments (LCAs) of food waste-to-BSF conversion systems report a relatively low global warming potential (GWP) of 17.36 kg CO_2_ per ton of functional unit. Notably, the pretreatment processes such as hydrothermal treatment, ionization, pulsed electric field discharge, and microbial treatment are identified as the primary contributors to emissions, rather than larval rearing itself. However, some LCA studies have also indicated that, under certain conditions, BSF-derived proteins may generate higher CO_2_ emissions per kilogram than high-yield conventional crops [44,164]. For example, one study found that about 41% of feed carbon was incorporated into larval body mass, while only 28.5% was lost as CO_2_ and negligible amounts as CH_4_ during a 7-day treatment, vs. slower microbial decomposition over 45 days, which lost nearly 48.6% of carbon to the atmosphere [333].

Economic analysis reveals that marginal viability at present mid-scale plants may require EUR 1–2 million in capital investment, and profitability depends on access to virtually free substrates and diversified revenue streams (meal, oil, frass, chitin, AMPs). Present markets for BSF products, often positioned as premium aqua-feed or pet food ingredients, limit volume and heighten financial risk, further compounded by regulatory uncertainty and nascent co-product markets [15,51]. Policy interventions, such as direct subsidies, carbon credits for waste valorization, or public procurement mandates for insect-fed animal products, could lower barriers, as could bio-refinery linkages (e.g., algae cultivation alongside the BSF) that valorize energy and material streams. Rigorous, system-specific LCAs and techno-economic studies that include all co-products and energy flows are essential to identify carbon and cost “hotspots” and guide process integration, while robust quality-management systems will be critical to build buyer confidence and drive down prices over time.

**Table 6:** BSF application key challenges with potential solutions.

Application	Key Challenges	Potential Solutions
Waste Management	Variable feed quality, contaminant carry-over, high-moisture frass	Sort and blend substrates, rigorous contaminant testing, frass composting/drying
Biodiesel/Oil	Diet-dependent lipid, energy-intensive extraction	Use high-fat wastes, green extraction methods, valorize protein/chitin coproducts
Animal Feed	Regulatory gaps, nutrient variability, allergenicity	Advocate insect-specific limits, routine safety assays, defatting/fractionation
AMP Recovery	Low yields, peptide instability, unclear regulations	Enhance expression (breeding/probiotics), advanced purification and encapsulation, early regulatory engagement
Bioremediation	Unpredictable metabolites, residue risks	Closed-loop reactors, coupled secondary treatments, pollutant fate studies
Automation and Technology	Manual controls, labor-intensive sorting	IoT-enabled rearing, vision-guided harvesters, modular, open-source hardware

## 7. Conclusions

The evidence reviewed herein underscores the BSF as a uniquely capable bioconversion agent whose intrinsic life-history traits, genomic adaptations, and microbial symbioses collectively enable rapid, high-efficiency transformation of heterogeneous organic wastes into nutrient-dense biomass and value-added byproducts. Critically, BSF systems redistribute up to 60% of substrate carbon into larval biomass, substantially lowering net CO_2_ emissions compared to composting or vermicomposting, while simultaneously attenuating pathogen loads and antibiotic resistance genes. Yet, these performance metrics co-exist with persistent obstacles: substrate variability yields inconsistent nutritional profiles, and contaminant burdens are unclear; regionally fragmented regulations impose burdensome testing without clear insect-specific safety thresholds, and high capital and energy demands constrain economic viability beyond pilot scales.

Moving forward, targeted innovations must focus on (1) feedstock standardization and pretreatment, integrating sensor-guided sorting or microbial consortia to buffer substrate heterogeneity; (2) regulatory harmonization, establishing validated contaminant limits and welfare guidelines to de-risk industrial investment; and (3) process intensification, leveraging automation, green extraction technologies, and life cycle-driven site co-location to minimize energy footprints. Interdisciplinary research bridging genomics, systems engineering, and policy analysis will be essential to translate laboratory successes into resilient, scalable BSF platforms. With continued technological innovation, operational scaling, and supportive policy frameworks, BSF-based systems could contribute significantly to circular bio-economies, closing nutrient loops, mitigating greenhouse gases, and generating sustainable protein and bio-product streams at industrial scales.

## Supplementary Materials

The following supporting information can be downloaded at https://www.mdpi.com/article/10.3390/insects16080750/s1, Table S1: Global Black Soldier Fly larvae production companies.

## Abbreviations

The following abbreviations are used in this manuscript:

BSF	Black Soldier Fly
BSFL	Black Soldier Fly larvae
AMPs	Antimicrobial peptides
ARGs	Antibiotic Resistance Genes
MAPK	Mitogen-activated protein Kinase
NF-κB	Nuclear Factor kappa-light-chain-enhancer of activated B cells
PUFAs	Polyunsaturated Fatty Acids
CLA	Conjugated Linoleic Acid
VOCs	Volatile Organic Compounds
CFP	Catalytic Fast Pyrolysis
NPK	Nitrogen, Phosphorus, and Potassium (used in the context of fertilizers)
IoT	Internet of Things
DNA	Deoxyribonucleic Acid
RNA	Ribonucleic Acid
WDG	Wet distiller’s grains
SMS	Spent mushroom substrate
CRISPR	Clustered Regularly Interspaced Short Palindromic Repeats
Gb	Gigabase (one billion base pairs)
ACE inhibitors	Inhibit angiotensin-converting enzyme
IPIFF	International Platform of Insects for Food and Feed
DPP-IV-inhibitory	Inhibits dipeptidyl peptidase-IV
AAFCO	Association of American Feed Control Officials
CO_2_-eq	Carbon dioxide equivalent
EFSA	European Food Safety Authority
FSM	Food Safety Modernization Act
LCA	Life cycle assessment
EU	European Union
